# A Case of Bilateral Cryptococcal Olecranon Bursitis

**DOI:** 10.7759/cureus.64449

**Published:** 2024-07-13

**Authors:** Saverio Francesco Bianco, Andrea Noah Paris, Nicole Galdes, Bernice Bianco

**Affiliations:** 1 Orthopaedics and Trauma, Mater Dei Hospital, Msida, MLT; 2 Surgery, Mater Dei Hospital, Msida, MLT; 3 Obstetrics and Gynaecology, Mater Dei Hospital, Msida, MLT

**Keywords:** bursectomy, cryptococcus neoformans, fungal, septic, bursitis, olecranon

## Abstract

Non-septic olecranon bursitis (NSOB) is the inflammation of the olecranon bursa, which is usually self-limiting with aseptic clinical manifestations. NSOB can be idiopathic or secondary to repetitive trauma or rheumatological conditions. Septic olecranon bursitis (SOB) is usually caused by bacterial skin colonisers, such as staphylococci and streptococci, and patients tend to present with systemic symptoms requiring medical and/or surgical interventions.

Herein we present a case of disseminated cryptococcal infection stemming from bilateral septic olecranon bursitis in a previously healthy immunocompetent 24-year-old female. Fluid cultures were positive for *Cryptococcus neoformans*. Patient underwent bilateral olecranon bursectomy, washouts and debridement. Moreover, the patient was started on long-term intravenous amphotericin B and later switched to oral flucytosine and fluconazole with good effect. Patient had good clinical outcomes at one-year follow-up.

SOB secondary to unusual pathogens, such as *Cryptococcus neoformans*, is a rare occurrence, and tends to affect immunocompromised individuals. The clinical course of such infections has shown to be subtle and insidious, which in turn hinders the diagnosis and leads to inappropriate treatment administration. Early follow-up and consideration of these organisms, together with appropriate discussion with microbiologists and/or infectious disease teams is crucial to reduce long-term morbidity and mortality.

## Introduction

Olecranon bursitis is a common condition in which patients present with elbow discomfort, swelling and limited range of movement. It is commonly classified into non-septic olecranon bursitis (NSOB) or septic olecranon bursitis (SOB) [1].

The aetiology of NSOB can be idiopathic, mechanical, or associated with autoimmune diseases. It is prevalent in men, especially manual workers with repetitive trauma to the elbow, with a peak incidence between the ages of 40 and 60 years [1]. Conservative management with regular anti-inflammatory medication, support devices (elbow padding + compression), activity modification, and early physiotherapy is the mainstay of treatment [1]. However, if symptoms recur, the chronic inflammatory response can lead to permanent epithelial damage with possible bone involvement. Therefore, in such cases, aspiration, infiltration, and surgical intervention (bursectomy) might need to be considered [1].

On the other hand, SOB is an uncommon aetiology and presents with fever, fluctuant swelling, and systemic upset. The commonest causative pathogens are bacteria, namely *Staphylococcus aureus* and other skin commensals [2]. In these circumstances, intravenous antibiotic therapy is the mainstay of treatment. Bursectomy, along with washout and drainage, is recommended for severe, refractory, or recurrent cases [2]. Current literature and clinical evidence on management of septic olecranon bursitis due to fungal infections are limited, and cases involving *Cryptococcus neoformans* are even rarer, hence this case report.

## Case presentation

A 24-year-old female from Pakistan was referred to the emergency department at Mater Dei Hospital due to worsening bilateral elbow swelling and erythema, with the right elbow more affected than the left. A week earlier, she had consulted her private doctor for the same symptoms and was prescribed a seven-day course of co-amoxiclav, which had little to no effect.

On examination, bilateral erythema and swelling of both elbows were noted, with the right elbow appearing to be more severely affected. Limited range of movement was observed in both elbows, again more pronounced on the right. Both elbows were held in fixed flexion due to significant discomfort and tenderness. Systematic examination revealed no involvement of other bursae and no additional signs or abnormalities.

**Figure 1:**
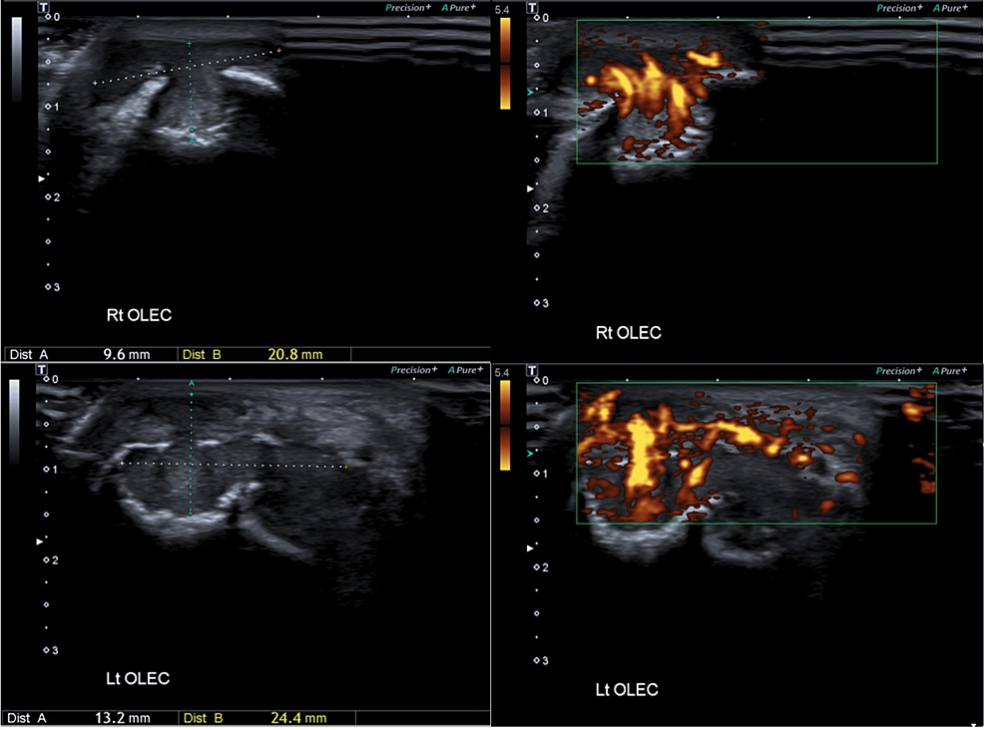
Ultrasound Elbow: There is evidence of abnormal soft tissue thickening in the olecranon regions bilaterally, worse on the left. The soft tissue swelling measures 13 x 25 x 33 mm on the left and 10 x 21 x 19 mm on the right. Bilateral erosions of the olecranon are noted. There is severe increased vascularity in the described soft tissue thickening bilaterally.

A bilateral elbow ultrasound (US) was performed, and an abnormal soft tissue thickening was observed in the olecranon regions bilaterally, worse on the left. A soft tissue swelling measuring 13 x 25 x 33 mm was observed at the left elbow and another soft tissue swelling measuring 10 x 21 x 19 mm on the right. Bilateral erosions of the olecranon were noted. There was severe increased vascularity in the described soft tissue thickenings bilaterally (Figure 1).

An aspirate was obtained from her left bursa and was sent for Microscopy, Culture & Sensitivity (MC&S).* Cryptococcus neoformans* was cultured from the fluid (Table 1).

**Table 1:** Left elbow aspirate Microscopy, Culture & Sensitivity (MC&S) result. S = Sensitive, R = Resistant

Mycology Investigations	
*Cryptococcus neoformans* cultivated	
Antifungal	Sensitivities
5 Flurocytosine	S
Amphotericin B	S
Anidulafungin	R
Caspofungin	R
Fluconazole	S
Itraconazole	S
Micafungin	R
Voriconazole	S

Since the right elbow was clinically worse than the left, it was decided to treat the right elbow surgically and attempt a more conservative medical management for the left elbow. Subsequently, the patient underwent a bursectomy and debridement of her right posterior olecranon. Separate tissue and bone specimens, obtained during the procedure, were sent for MC&S and *Cryptococcus neoformans* was once again cultivated from these specimens. As per microbiology and infectious disease advise, the patient was started on long-term intravenous amphotericin B (Ambisome).

**Figure 2:**
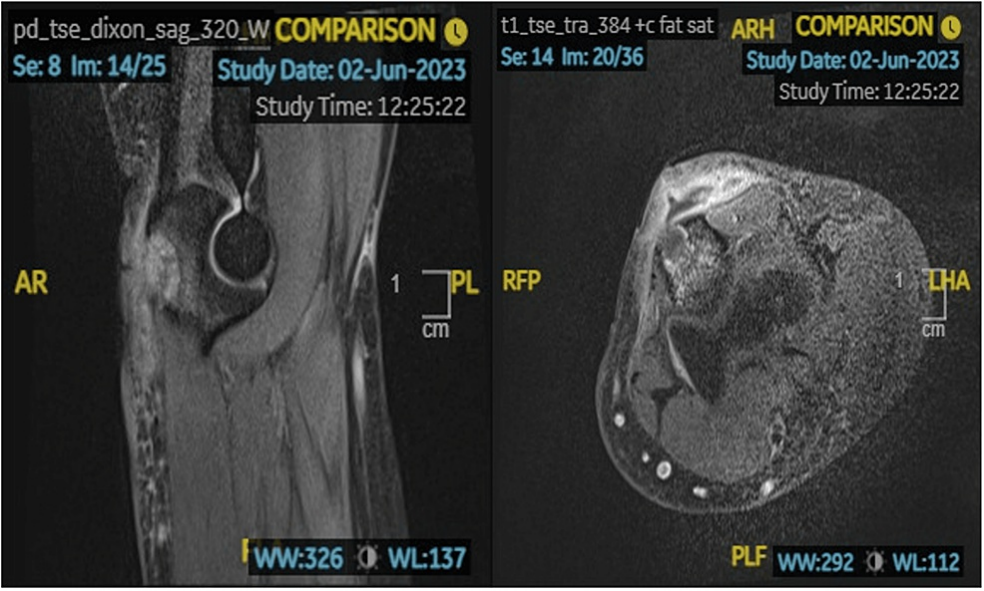
MR Right Elbow: Subcutaneous rim enhancing collection with surrounding soft tissue enhancement in the region of the olecranon bursa measuring approximately 23mm (trans) x 24mm (CC) x 12mm (AP), which erodes into the olecranon. Surrounding bone marrow oedema like signal change and enhancement noted. Tiny elbow joint effusion. No pericapsular oedema, synovitis, or periarticular bone marrow changes to suggest septic arthritis. AP, anteroposterior; trans, transverse; CC, craniocaudal

During her admission, the patient developed fever, accompanied by dizziness and increased lethargy. A lumbar puncture was performed, which excluded cryptococcal meningitis. The case was discussed with a local upper limb specialist who advised magnetic resonance (MR) imaging to rule out any fluid or collections within the joint, which would require proper washout. MR imaging of her right elbow excluded features consistent with septic arthritis (Figure 2).

**Figure 3:**
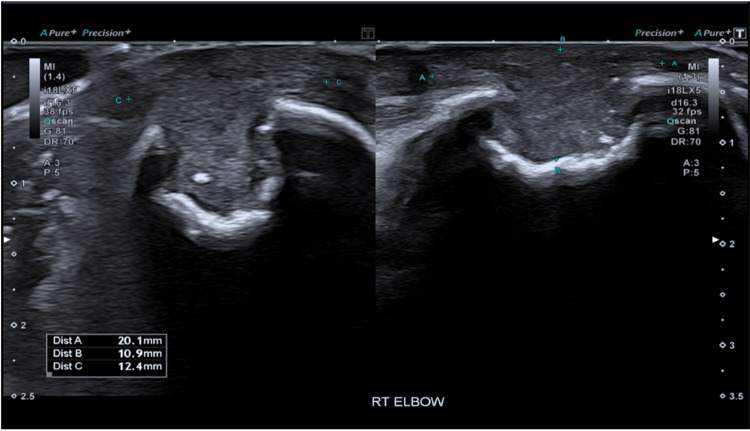
Ultrasound Right Elbow: The superficial component of the previously described olecranon bursa is essentially collapsed. The component eroding into the olecranon is still evident. There is no surrounding hyperaemia to suggest active inflammatory changes.

Despite being on adequate IV anti-fungal therapy, the left elbow swelling worsened and a yellowish discharge was observed during a follow-up clinical examination. On examination, the right elbow was now clinically unremarkable. A bilateral elbow US was repeated. The right elbow US revealed that the superficial component of the previously described olecranon bursa had essentially collapsed. The component eroding into the olecranon however was still evident. There was no surrounding hyperaemia to suggest active inflammatory changes (Figure 3).

**Figure 4:**
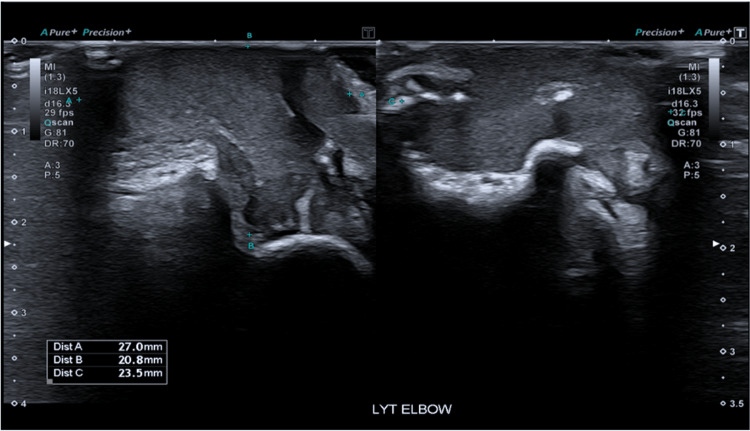
Ultrasound Left Elbow: Persistent 27mm x 21mm x 23mm superficial collection adjacent to and eroding into the left olecranon with marked surrounding inflammatory change. No elbow joint effusion.

However, the left elbow US showed a persistent superficial collection measuring 27mm x 21mm x 23mm adjacent to and eroding into the left olecranon with marked surrounding inflammatory change. No elbow joint effusion was observed (Figure 4).

Given the above clinical and radiological findings, it was agreed to proceed with incision and drainage of the left olecranon bursa. Following the procedure, IV amphotericin B was administered for a total of four weeks. Patient was reviewed again on an outpatient basis, with regular serial blood tests. At the first clinic visit, it was noted that *Staphylococcus aureus* was cultivated from the left elbow, however, this was disregarded as a coloniser. Thereafter, inflammatory markers returned to normal levels and remained stable throughout the follow-up period.

Eventually, the patient was switched to oral flucytosine for a duration of one month and then started on oral fluconazole. At the three-month follow-up appointment, the patient was noted to be well. The bilateral elbow wounds had healed adequately with full range of movement and no evidence of recurrence. The patient was reviewed by the immunology team who advised further immunological testing including nitro blue tetrazolium chloride (NBT) which was normal, immunoglobulin (Ig) E-7 which was also noted to be normal, Th17 T-cells, anti-cytokine antibody testing, and other genetic tests.

Following a discussion at the local multi-disciplinary team meeting with specialist advice from Sanford (United States), it was agreed to treat as cryptococcal meningitis for another 12 months even though there was no evidence of central nervous system (CNS) involvement. At the one-year follow-up, the patient was found to be clinically well. The bilateral elbow wounds had healed well and on examination both elbows were unremarkable with no signs of recurrence. The patient, however, was still concerned of occasional intermittent mild discomfort in the left elbow. The patient will continue treatment with fluconazole and will be reviewed by the infectious disease team with further follow-up imaging as needed.

## Discussion

The olecranon bursa is a synovial membrane located posterior to the elbow joint. Its superficial position and limited vascular supply make it susceptible to trauma, infection, and inflammation [3]. Olecranon bursitis is a clinical diagnosis and in NSOB cases, management is usually conservative and involves rest, ice, compression, and early physiotherapy [3]. However, SOB is a more challenging situation, and diagnostic tests are essential, considering the possibility of disseminated infection [2].

The common pathogens causing septic olecranon bursitis are bacteria such as staphylococci or streptococci which migrate transcutaneously through repetitive minor trauma [2,3]. Most cases of SOB are secondary to direct inoculation, however haematogenous spread has also been reported [4].

Fungal olecranon bursitis is a rare presentation. Early diagnosis can be challenging as it manifests similar symptoms to a bacterial infection but tends to have a more indolent course. This similarity can lead to delays in accurate diagnosis and inappropriate treatment, potentially resulting in long-term morbidity.

The fungi most commonly reported in medical literature as causing SOB are the *Candida* and *Penicillium* species, *Aspergillus terreus*, and *Phialophora richardsiae* [5]. Only one case of septic olecranon bursitis secondary to *Cryptococcus neoformans* has been reported in medical literature. Farr and Wright (1992) reported a case of SOB caused by *Cryptococcus neoformans* in a 47-year-old gentleman with liver cirrhosis [6]. In contrast, our case reports a healthy immunocompetent young lady who presented with bilateral olecranon bursitis.

*Cryptococcus neoformans* is an encapsulated opportunistic fungal organism that usually inhabits debris around pigeon roosts and soil contaminated with pigeon droppings [7] and it is generally accepted that the organism enters the host via the respiratory route [8]. Most cryptococcal infections in both immunocompromised, as well as immunocompetent patients, occur in the lungs. However, *Cryptococcus neoformans* can disseminate haematologically from the lungs to the other organs. Dissemination to the meninges can result in cryptococcal meningitis or meningoencephalitis, which may be fatal [7].

The simultaneous involvement of both olecranon bursae suggests hematogenous dissemination from the lungs as the likely source of infection, rather than transcutaneous migration. Apart from the bilateral olecranon bursae, there was no evidence of central nervous system or organ involvement. The reasons for the specific involvement of both olecranon bursae and the lack of any other affected bursae or organs remain unclear.

There are currently no established guidelines for managing fungal bursitis. Typically, initial treatment involves intravenous or oral antifungal medications, sometimes combined with surgical intervention such as bursectomy. However, consensus is lacking on when surgery should be performed - whether to go for early bursectomy at diagnosis or after a trial of antifungal therapy.

In our case, immediate surgical intervention was undertaken for right olecranon bursa, while the left elbow received a trial of intravenous amphotericin B. Despite four weeks on IV amphotericin B, symptoms persisted, necessitating surgical debridement of the left olecranon bursa. At the one-year follow-up, the right elbow, treated promptly with bursectomy, had fully recovered, whereas the left elbow still experienced intermittent pain. This underscores the potential benefit of early surgical intervention, specifically bursectomy, in fungal suppurative olecranon bursitis to mitigate long-term morbidity.

## Conclusions

Fungal septic bursitis due to *Cryptococcus neoformans* is exceptionally rare and poses significant diagnostic challenges. Clinicians should maintain a high index of suspicion in immunocompromised patients presenting with olecranon bursitis. However, it is also essential to consider this diagnosis in immunocompetent patients who do not respond to empirical treatment. Although bacterial septic bursitis normally responds well to antibiotic therapy, fungal cryptococcal bursitis may not respond adequately to antifungals and early bursectomy should be considered in the management of such cases to prevent long-term morbidity.

## References

[1] Baumbach SF, Lobo CM, Badyine I, Mutschler W, Kanz KG (2014). Prepatellar and olecranon bursitis: literature review and development of a treatment algorithm. Arch Orthop Trauma Surg.

[2] Charret L, Bart G, Hoppe E (2021). Clinical characteristics and management of olecranon and prepatellar septic bursitis in a multicentre study. J Antimicrob Chemother.

[3] angia J, Rizvi TJ (2024). Olecranon bursitis. StatPearls [Internet].

[4] Truong J, Mabrouk A, Ashurst JV (2024). Septic bursitis. StatPearls [Internet].

[5] Gamarra-Hilburn CF, Rios G, Vilá LM (2016). Olecranon bursitis caused by Candida parapsilosis in a patient with rheumatoid arthritis. Case Rep Rheumatol.

[6] Farr RW, Wright RA (1992). Cryptococcal olecranon bursitis in cirrhosis. J Rheumatol.

[7] Zhao Y, Ye L, Zhao F (2023). Cryptococcus neoformans, a global threat to human health. Infect Dis Poverty.

[8] Spahr J, Weiner DJ, Stokes DC, Kurland G (2012). Pulmonary disease in the pediatric patient with acquired immunodeficiency states. Kendig & Chernick's Disorders of the Respiratory Tract in Children (Eighth Edition).

